# Risk factors for early mortality and severe intraventricular hemorrhage in extremely preterm infants with gestational age <28 weeks: a retrospective case-control study

**DOI:** 10.3389/fped.2025.1715767

**Published:** 2026-01-15

**Authors:** Ziqi Wu, Yimeng Zhao, Ruifeng Tian, Sicong Peng, Qin Liu, Shiwen Xia, Yi Zhang

**Affiliations:** 1 Department of Neonatology, Maternal and Child Health Hospital of Hubei Province, Tongji Medical College, Huazhong University of Science and Technology, Wuhan, Hubei, China; 2 Department of Ultrasound, Maternal and Child Health Hospital of Hubei Province, Tongji Medical College, Huazhong University of Science and Technology, Wuhan, Hubei, China

**Keywords:** extremely preterm infants, outcome, death, intraventricular hemorrhage, risk factor

## Abstract

**Introduction:**

Extremely premature infants (EPIs) are at significant risk for early mortality and severe intraventricular hemorrhage. This study aimed to investigate the risk factors associated with early mortality and severe intraventricular hemorrhage in EPIs with a gestational age of less than 28 weeks and to evaluate the predictive value of these risk factors in determining adverse outcomes.

**Methods:**

A retrospective analysis was conducted on clinical data from EPIs admitted to the Neonatal Intensive Care Unit at Maternal and Child Health Hospital of Hubei Province between January 2019 and December 2024. Infants were categorized into two groups based on their early outcomes: an adverse outcome group (*n* = 110) and a favorable outcome group (*n* = 183). Binary logistic regression analysis was used to identify high-risk factors for adverse outcomes in EPIs, and receiver operating characteristic (ROC) curve analysis was performed to assess the predictive value of these factors.

**Results:**

This study revealed that the maximum vasoactive-inotropic score (Max VIS) (*OR*: 1.136, 95% *CI*: 1.070, 1.216) and middle cerebral artery resistance index (MCA-RI) (*OR*: 450.489, 95%*CI*: 36.163, 5,611.780) and vaginal delivery (*OR*: 3.684, 95%*CI*: 2.005, 6.768) were independent risk factors for adverse outcomes in EPIs, while gestational age was a protective factor (OR: 0.568, 95% CI: 0.415, 0.778). ROC curve analysis indicated that Max VIS > 9.5, MCA-RI > 0.81, vaginal delivery, and small gestational age had predictive value for adverse outcomes in EPIs (*P* < 0.05), with area under the curves (AUC) of 0.680 (95% *CI*: 0.615, 0.745), 0.693 (95%*CI*: 0.628, 0.758), 0.653 (95% *CI*: 0.588, 0.718), and 0.660 (95% CI: 0.275, 0.404), respectively. The combination of all four factors yielded the highest predictive performance, with an AUC of 0.833 (95%*CI*: 0.783, 0.883), sensitivity of 72.7%, and specificity of 81.4%.

**Conclusion:**

Elevated Max VIS, increased MCA-RI, vaginal delivery, and small gestational age are independent risk factors for early mortality and severe intraventricular hemorrhage in EPIs. Each is a valuable predictor of adverse outcomes, and their combination demonstrates the highest predictive value, providing significant clinical reference for the early management of these high-risk neonates.

## 1 Introduction

Preterm birth is a significant global issue affecting newborn health. With the continuous advancement of perinatal medical technologies, the number of extremely premature infants (EPIs) has been increasing annually. However, due to the extreme immaturity of their organ systems, EPIs still face high mortality rates and a high incidence of severe complications. The primary clinical challenges for EPIs include respiratory distress syndrome, intraventricular hemorrhage (IVH), bronchopulmonary dysplasia, necrotizing enterocolitis, retinopathy of prematurity, and postnatal growth restriction (1, 2). Among these, early mortality and severe intraventricular hemorrhage (sIVH) are the most critical adverse outcomes impacting the prognosis of EPIs. Studies have shown that the risk of death in EPIs is inversely related to gestational age; the smaller the gestational age, the higher the mortality rate (3). The incidence of sIVH, a severe neurological complication in EPIs, increases significantly with decreasing gestational age. More critically, sIVH not only directly threatens the lives of affected infants but also leads to varying degrees of neurodevelopmental sequelae in survivors, including cerebral palsy, intellectual disability, epilepsy, and visual and auditory impairments, which severely affect long-term quality of life (4, 5).

These adverse outcomes impose a heavy psychological and economic burden on families and place significant pressure on healthcare resources. Although clinical management of EPIs continues to improve, the risk factors for early mortality and severe outcomes such as sIVH remain incompletely understood. Therefore, investigating the key risk factors influencing adverse outcomes in EPIs and establishing effective predictive models are of significant clinical value for improving clinical decision-making and enhancing the quality of life for these infants.

This study aims to systematically investigate the risk factors influencing early adverse outcomes in EPIs through retrospective analysis of clinical data and to evaluate the predictive value of these risk factors for adverse outcomes, providing scientific evidence for clinical practice.

## 2 Methods

### 2.1 Study design

**Figure 1:**
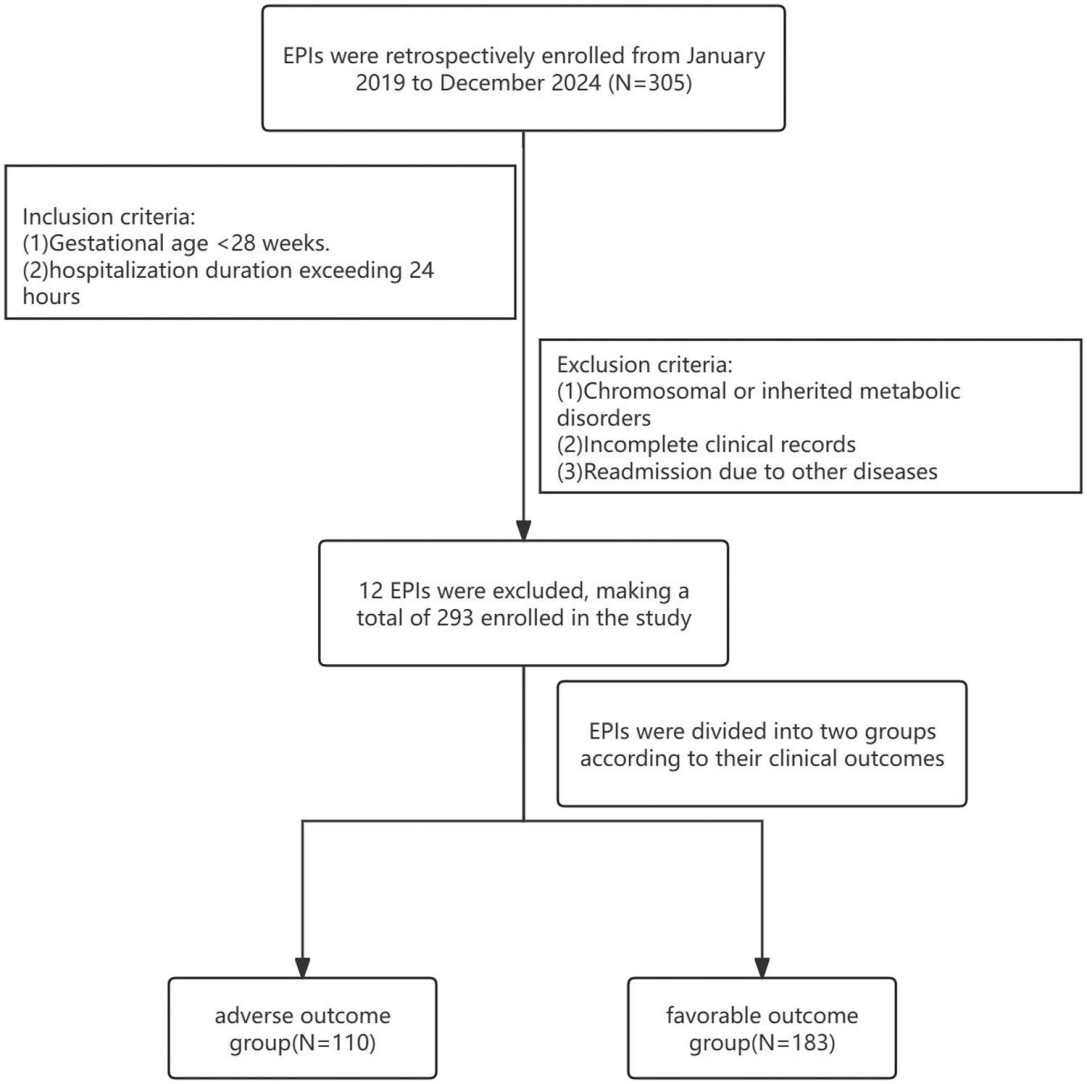
The protocol and procedure for this study.

This retrospective study included EPIs admitted to the Neonatal Intensive Care Unit at Maternal and Child Health Hospital of Hubei Province between January 2019 and December 2024. Infants were categorized into two groups based on their clinical outcomes during hospitalization: the adverse outcome group and the favorable outcome group. Inclusion criteria included: gestational age <28 weeks and hospitalization duration exceeding 24 h. Exclusion criteria were: (1) sIVH was already present on the first cranial ultrasound examination within 24 h after birth; (2) chromosomal or inherited metabolic disorders; (3) incomplete clinical records; (4) readmission due to other diseases (e.g., pneumonia, jaundice, anemia) (Figure 1).

### 2.2 Data collected

Clinical data were collected from all study participants, including the following categories: (1) Neonatal baseline characteristics: sex, gestational age at birth, birth weight, small for gestational age (SGA), *in vitro* fertilization, twin or multiple births, vaginal delivery, and intrauterine distress. (2) Maternal prenatal history: maternal comorbidities during pregnancy (diabetes, hypertension, infection), cervical cerclage, use of prenatal magnesium sulfate, use of antenatal corticosteroids, prolonged rupture of membranes (>18 h), placental abruption, meconium-stained amniotic fluid, and umbilical cord abnormalities (cord torsion, true knot, or thin cord). (3) Neonatal clinical data within the first week of life: delivery room resuscitation (endotracheal intubation, chest compressions, epinephrine administration), severe asphyxia after birth, moderate-to-severe hypothermia within the first hour of life, administration of pulmonary surfactant within 2 h after birth, blood gas pH within 12 h of life, early-onset sepsis, pulmonary hemorrhage, hemodynamically significant patent ductus arteriosus, maximum vasoactive-inotropic score (Max VIS) within 24 h after birth, and middle cerebral artery resistance index (MCA-RI) measured within 24 h of life.

### 2.3 Diagnostic criteria

Adverse outcomes (6, 7) were defined as death within 24 h after family-signed withdrawal of life-sustaining treatment, in-hospital mortality despite resuscitation efforts, and/or the occurrence of sIVH within the first week of life. IVH was classified according to the Papile grading system: grade I: unilaterally or bilaterally subependymal hemorrhage; grade II: intraventricular hemorrhage without ventricular dilatation; grade III: intraventricular hemorrhage with ventricular dilatation; grade IV: ventricular dilatation with periventricular white matter injury or hemorrhagic infarction. grades I–II were considered mild, and grades III–IV were considered severe (8). SGA was defined as birth weight below the 10th percentile for gestational age, according to the 2013 Fenton growth charts for preterm infants (9). Severe asphyxia was defined as: Apgar score ≤3 at 1 min or ≤5 at 5 min, and/or umbilical artery pH <7.0 (10). Moderate to severe hypothermia was defined as an admission temperature ≤35.9°C (11). Early-onset sepsis was defined as clinical signs of infection, abnormal nonspecific laboratory tests, or positive microbiological findings within the first 72 h of life (12). Hemodynamically significant patent ductus arteriosus was diagnosed based on the following criteria:(1) left-to-right or bidirectional shunting confirmed by echocardiography; (2) left atrium to aortic root ratio >1.4; (3) ductal diameter >1.5 mm; (4) presence of at least one of the following clinical signs: cardiac murmur, bounding pulse, tachycardia, increased precordial activity, widened pulse pressure, or worsening respiratory status (13, 14). VIS = dopamine dose [μg/(kg·min)] + dobutamine dose [μg/(kg·min)] + 10 × milrinone dose [μg/(kg·min)] + 100 × epinephrine dose [μg/(kg·min)] + 100 × norepinephrine dose [μg/(kg·min)] + 10 000 × vasopressin dose [U/(kg·min)] (15).

### 2.4 Sample size estimation

Since the various factors in binary logistic regression are interdependent and influence each other, it is difficult to make an accurate prediction of the sample size. A common guideline suggests that the sample size should be 5, 10, or even 20 times the number of variables. In this study, the most stringent criterion was applied, requiring the total sample size to be no less than 20 times the number of variables (16).

### 2.5 Statistical analysis

Continuous variables with normal distribution were presented as mean ± standard deviation (mean ± SD), and group comparisons were performed using the independent samples *t*-test. Non-normally distributed continuous variables were presented as median with interquartile range [*M* (*P*25, *P*75)], and group comparisons were conducted using the Mann–Whitney *U* test. Categorical variables were presented as counts and percentages (%), and comparisons between groups were performed using the chi-square (*χ*^2^) test or Fisher's exact test, as appropriate.

To identify independent risk factors for adverse outcomes in EPIs, binary logistic regression analysis with a stepwise method was performed. The predictive performance of significant risk factors was evaluated using receiver operating characteristic (ROC) curves. The area under the curve (AUC), sensitivity, and specificity were calculated. The optimal cutoff value was determined as the value yielding the maximum sum of sensitivity and specificity.

Statistical analysis was performed using SPSS 26.0. A *p*-value < 0.05 was considered statistically significant.

## 3 Results

### 3.1 Early adverse outcomes in EPIs

A total of 293 EPIs with a gestational age <28 weeks were included in this study. Among them, 91 infants died, including 81 cases of death following family-signed withdrawal of life-sustaining treatment and 10 cases of in-hospital mortality despite resuscitation efforts.

When stratified by gestational age at birth, the mortality rates for EPIs born at <24, 24, 25, 26, and 27 weeks were 60%, 45.5%, 45.7%, 39.5%, and 21.7%, respectively. The corresponding incidence rates of sIVH were 20%, 54.5%, 37.1%, 18.5%, and 11.2%. The differences among the five groups were statistically significant (*P* < 0.05) (Table 1).

**Table 1:** Early adverse outcomes in extremely preterm infants according to gestational age and birth weight groups.

Gestational age group (weeks)	Death *N* = 91	sIVH *N* = 53	Birth weight group (g)	Death *N* = 91	sIVH *N* = 53
<24	3 (60%)^a^	1 (20%)^a^	<500	4 (100%)^b^	–
24–24^+6^	5 (45.5%)^a^	6 (54.5%)^a^	500–749	19 (47.5%)^b^	16 (40%)^b^
25–25^+6^	16 (45.7%)^b^	13 (37.1%)^b^	750–999	42 (30.2%)^a^	24 (17.3%)^a^
26–26^+6^	32 (39.5%)^a^	15 (18.5%)^a^	1 000–1 249	26 (24.5%)^a^	13 (12.3%)^a^
27–27^+6^	35 (21.7%)^b^	18 (11.2%)^b^	1 250–1 499	0^a^	0^a^
*χ* ^2^	–	–	*χ* ^2^	–	–
*P*	0.002*	0.001*	*P*	0.002*	0.005*

sIVH, severe intraventricular hemorrhage.

a,b Significant differences between groups within the same category.

* *P*-value calculated using Fisher's exact test.

When stratified by birth weight, the mortality rates for EPIs weighing <500 g, 500–749 g, 750–999 g, 1,000–1,249 g, and 1,250–1,499 g were 100%, 47.5%, 30.2%, 24.5%, and 0%, respectively. The incidence rates of sIVH in the 500–749 g, 750–999 g, 1,000–1,249 g, and 1,250–1,499 g groups were 40%, 17.3%, 12.3%, and 0%, respectively. The differences among the groups were also statistically significant (*P* < 0.05) (Table 1).

### 3.2 Analysis of causes of death in EPIs

In this study, among the in-hospital deaths, cardiogenic shock and grade III-IV respiratory distress syndrome (RDS) were the primary causes of mortality, accounting for 40% and 30%, respectively. For infants who died after the withdrawal of treatment, the main causes of death were severe IVH (sIVH) and grade III-IV RDS, constituting 46.9% and 37%, respectively (Table 2).

**Table 2:** Analysis of causes of death in extremely premature infants.

Group	Number of cases	III-IV RDS (%)	sIVH (%)	Pulmonary hemorrhage (%)	EOS/septic shock (%)	Cardiogenic shock (%)
In-hospital death	10	3 (30)	0	1 (10)	2 (20)	4 (40)
Death after giving up treatment	81	30 (37)	38 (46.9)	4 (4.9)	4 (4.9)	5 (6.2)

RDS, respiratory distress syndrome; sIVH, severe intraventricular hemorrhage; EOS, early-onset sepsis.

### 3.3 Univariate analysis of early adverse outcomes in EPIs

A total of 293 EPIs were included in this study, of whom 110 (37.5%) were assigned to the adverse outcome group and 183 (62.5%) to the favorable outcome group.The median gestational ages were 26.3 (25.4, 27.2) weeks in the adverse outcome group and 27.1 (26.4, 27.4) weeks in the favorable outcome group. The median birth weights were 900 (720, 1,000) g and 980 (850, 1,080) g, respectively. No significant differences were observed between the two groups in terms of male sex, SGA, *in vitro* fertilization, intrauterine distress, blood pH within 12 h after birth, or maternal pregnancy-related conditions (all *P* > 0.05).

Compared with the favorable outcome group, the adverse outcome group had significantly lower gestational age and lower birth weight. The adverse outcome group also showed higher proportions of multiple births, vaginal delivery, intubation in the delivery room, severe asphyxia, and pulmonary hemorrhage within the first postnatal week. In addition, the Max VIS and MCA-RI within 24 h after birth were significantly higher in the adverse outcome group (all *P* < 0.05) (Table 3).

**Table 3:** Univariate analysis of early adverse outcomes in extremely preterm infants.

Characteristics	Adverse outcome group *N* = 110	Favorable outcome group *N* = 183	*χ*^2^*/*z	*P*-value
Sex, male, *n* (%)	62 (56.4%)	108 (59%)	0.198	0.656
Gestational age, weeks	26.3 (25.4,27.2)	27.1 (26.4,27.4)	−4.600	<0.001
Birth weight, gram	900 (720,1 000)	980 (850,1 080)	−3.382	0.001
SGA, *n* (%)	3 (2.7%)	2 (1.1%)	–	0.368^b^
*In vitro* fertilization, *n* (%)	38 (34.5%)	62 (33.9%)	0.014	0.907
Multiple births, *n* (%)	48 (43.6%)	58 (31.7%)	4.244	0.039
Fetal distress, *n* (%)	9 (8.2%)	17 (9.3%)	0.104	0.747
Vaginal delivery, *n* (%)	74 (67.3%)	67 (36.6%)	25.871	<0.001
Pregnancy-induced hypertension, *n* (%)	11 (10%)	17 (9.3%)	0.040	0.841
Gestational diabetes, *n* (%)	15 (13.6%)	38 (20.8%)	2.356	0.125
Perinatal infection, *n* (%)	28 (25.5%)	44 (24%)	0.074	0.786
Cervical cerclage, *n* (%)	14 (12.7%)	29 (15.8%)	0.534	0.465
Magnesium sulfate use, *n* (%)	40 (36.4%)	65 (35.5%)	0.021	0.884
Antenatal corticosteroid use, *n* (%)	71 (64.5%)	128 (69.9%)	0.919	0.318
Premature rupture of membranes >18 h, *n* (%)	18 (16.4%)	37 (20.2%)	0.670	0.413
Placental abnormalities, *n* (%)	29 (26.4%)	38 (20.8%)	1.221	0.269
Amniotic fluid meconium staining, *n* (%)	27 (24.5%)	50 (27.3%)	0.273	0.601
Umbilical cord abnormalities, *n* (%)	18 (16.4%)	29 (15.8%)	0.014	0.907
Intubation in delivery room, *n* (%)	96 (87.3%)	133 (72.7%)	8.573	0.030
Chest compressions in delivery room, *n* (%)	13 (11.8%)	14 (7.7%)	1.427	0.232
Epinephrine in delivery room, *n* (%)	7 (6.4%)	7 (3.8%)	0.973	0.324
Severe asphyxia, *n* (%)	26 (23.6%)	20 (10.9%)	6.489	0.011
Moderate to severe hypothermia, *n* (%)	29 (26.4%)	39 (21.3%)	0.984	0.321
pH < 7.2 within 12 h of birth, *n* (%)	20 (18.2%)	17 (9.3%)	1.360	0.224
PS administration within 2 h, *n* (%)	77 (70%)	129 (70.5%)	0.008	0.929
Early-onset sepsis, *n* (%)	33 (30%)	54 (29.5%)	0.008	0.929
Pulmonary hemorrhage, *n* (%)	30 (27.3%)	24 (13.1%)	9.161	0.002
hsPDA, *n* (%)	42 (38.2%)	80 (43.7%)	0.866	0.352
Maximum VIS within 24 h, points	5 (0,10)	4.5 (0,7.5)	−2.016	0.044
Middle cerebral artery RI value within 24 h	0.8 (0.68,1)^a^	0.73 (0.66,0.81)	−3.278	0.001

SGA, small for gestational age; PS, Pulmonary surfactant; VIS, Vasoactive-inotropic score; hsPDA, Hemodynamically significant patent ductus arteriosus; RI, Resistance index.

a The adverse outcome group had 99 cases with middle cerebral artery RI values.

b *P*-value calculated using Fisher's exact test.

### 3.4 Multivariate binary logistic regression analysis of early adverse outcomes in EPIs

A binary logistic stepwise regression model was constructed to analyze factors associated with early adverse outcomes in EPIs. The presence of adverse outcome was set as the dependent variable (yes = 1, no = 0), and variables that showed statistical significance in the univariate analysis were included as independent variables. These included gestational age Z-score, multiple births, vaginal delivery, intubation in the delivery room, severe asphyxia, pulmonary hemorrhage, Max VIS, and MCA-RI.

The regression analysis revealed that gestational age Z-score (*OR*: 0.568, 95% *CI*: 0.415, 0.778), vaginal delivery (*OR*:3.684, 95% *CI*: 2.005, 6.768), Max VIS (*OR*:1.136, 95% *CI*: 1.070, 1.216), MCA-RI (*OR*:450.489, 95% *CI*: 36.163,5 611.780) were independently associated with early adverse outcomes (*P* < 0.05) (Table 4).

**Table 4:** Multivariate binary logistic regression analysis of factors associated with adverse outcomes in extremely preterm infants.

Variable	B	SE	Wald *χ*^2^	*P*-value	OR	95% CI
Gestational age Z-score	−0.565	0.160	12.414	<0.001	0.568	[0.415, 0.778]
Multiple births	0.177	0.320	0.306	0.580	1.193	[0.637, 2.234]
Vaginal delivery	1.304	0.310	17.645	<0.001	3.684	[2.005, 6.768]
Intubation in delivery room	0.486	0.410	1.402	0.236	1.626	[0.727, 3.633]
Severe asphyxia	0.516	0.334	2.384	0.123	1.675	[0.870, 3.222]
Pulmonary hemorrhage	0.550	0.402	1.872	0.171	1.733	[0.788, 3.809]
Maximum VIS within 24 h	0.128	0.031	17.371	<0.001	1.136	[1.070, 1.216]
Middle cerebral artery RI value within 24 h	6.110	1.287	22.544	<0.001	450.489	[36.163, 5,611.780]
Constant	−7.392	1.117	43.805	<0.001	0.001	–

VIS, vasoactive-inotropic score; RI, resistance index.

### 3.5 Predictive value of maximum VIS, middle cerebral artery RI, vaginal delivery and gestational age Z-score for adverse outcomes in EPIs

ROC curve analysis indicated that Max VIS > 9.5, MCA-RI > 0.81 and vaginal delivery had predictive value for adverse outcomes in EPIs (*P* < 0.05), with area under the curves (AUC) of 0.680 (95%*CI*: 0.615, 0.745), 0.693 (95%*CI*: 0.628, 0.758), and 0.653 (95%*CI*: 0.588, 0.718), respectively. However, the gestational age Z-score was identified as a protective factor for EPIs, with an AUC of 0.660 (1–0.340) (95% *CI*: 0.275, 0.404).

**Figure 2:**
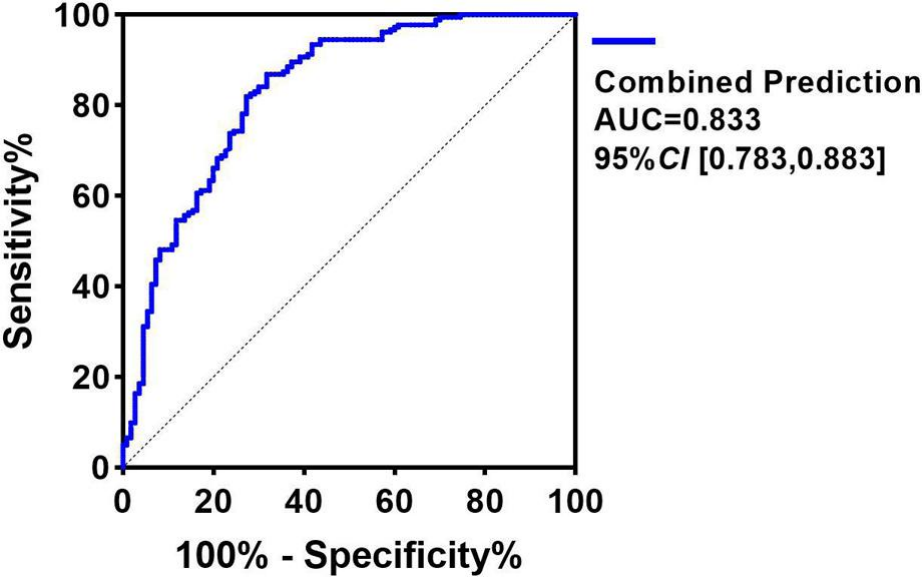
ROC curves showing the predictive value of maximum VIS, middle cerebral artery RI, and vaginal delivery for adverse outcomes in extremely preterm infants.

Notably, the combination of Max VIS, MCA-RI, vaginal delivery and gestational age Z-score demonstrated the highest predictive accuracy, with an AUC of 0.833 (95% *CI*: 0.783, 0.883), sensitivity of 72.7%, and specificity of 81.4% (Table 5; Figure 2).

**Table 5:** Predictive value of maximum VIS, middle cerebral artery RI, and vaginal deliveryforadverse outcomes in extremely preterm infant.

Variable	AUC	Optimal cut-off	95% CI	Sensitivity	Specificity
Maximum VIS	0.680	9.5	[0.615, 0.745]	0.609	0.749
Middle cerebral artery RI value	0.693	0.81	[0.628, 0.758]	0.624	0.727
vaginal delivery	0.653	0.5	[0.588, 0.718]	0.673	0.634
Gestational age Z-score	0.340	−0.55	[0.275–0.404]	0.503	0.678
Combined prediction	0.833	–	[0.783, 0.883]	0.727	0.814

VIS, vasoactive-inotropic score; RI, resistance index.

## 4 Discussion

EPIs with a gestational age of less than 28 weeks represent a challenging population in perinatal medicine, and their survival rates directly reflect the level of neonatal intensive care in a region. EPIs exhibit extreme immaturity of various organ systems and are prone to multiple complications, particularly sIVH, which significantly contributes to mortality risk (17, 18). This poses a major challenge for clinical management. Numerous epidemiological surveys and clinical studies have confirmed this grim reality, showing high mortality and severe complication rates across different countries and healthcare institutions. A French study reported that among EPIs aged 24–26 weeks, the in-hospital mortality rate and sIVH incidence were 29.2% and 21.8%, respectively, with a one-week postnatal mortality rate of 14.9% (19). Multicenter studies in China indicated EPIs mortality rates ranging from 40% to 62% (20, 21), and an IVH incidence of 13.7% (22). In our study, the overall mortality rate for EPIs was 31.1%, with smaller gestational ages associated with higher mortality rates; specifically, those under 24 weeks had a mortality rate as high as 60%. Additionally, the incidence of sIVH in our study was 18%, and it was the leading cause of death, accounting for 41.8% of mortality; both findings are, consistent with previous findings.

To investigate factors associated with the high mortality and high incidence of sIVH in EPIs, our binary logistic regression analysis showed that gestational age was a protective factor against adverse outcomes in EPIs. Smaller gestational age was associated with higher risk of adverse outcomes, with an OR of 0.568 (95% *CI*: 0.415, 0.778). Moreover, vaginal delivery was an independent risk factor for poor early outcomes (*OR*: 3.684, 95% *CI*: 2.005, 6.768). ROC curve analysis indicated that spontaneous vaginal delivery had moderate predictive ability for adverse outcomes, with an AUC of 0.653, sensitivity of 67.3%, and specificity of 63.4%, suggesting its potential utility in evaluating short-term prognosis. This finding is consistent with some previous studies (23, 24). EPIs have immature periventricular vascular structures and impaired cerebral autoregulation. During vaginal delivery, repeated uterine contractions, fetal head compression, and significant hemodynamic fluctuations may lead to transient surges in cerebral blood flow. This sudden increase can exceed the limited regulatory capacity of the developing cerebral vasculature, resulting in vascular rupture or ischemia-reperfusion injury, which may trigger the development of IVH (25). Previous evidence suggests that elective cesarean delivery may provide a more stable delivery process, reducing mechanical and hemodynamic stress, and potentially offering neuroprotective benefits for the immature brain (26).

While clinical decisions regarding mode of delivery depend not only on fetal condition but also on maternal status, availability of medical resources, and clinician experience, our findings highlight that delivery mode remains a modifiable perinatal factor with important implications for EPIs outcomes. Future studies are needed with larger sample sizes and more comprehensive perinatal variables to further examine the interaction between delivery mode and other risk factors, providing evidence-based guidance for optimal delivery planning in preterm infants.

The VIS is a crucial quantitative metric for assessing hemodynamic status in critically ill children. Studies have shown that high VIS often indicates severe circulatory failure or shock states, potentially caused by heart failure, sepsis, persistent pulmonary hypertension, and other pathological conditions. Consequently, higher VIS levels may correlate with an increased risk of in-hospital mortality (27). Moreover, elevated VIS is closely linked to severe IVH. On one hand, extensive use of vasoactive drugs can lead to dramatic blood pressure fluctuations, disrupting cerebral autoregulation mechanisms and inducing brain hemorrhages. On the other hand, initial hypotension-induced cerebral hypoxia followed by reperfusion injury during resuscitation exacerbates brain damage (28, 29).

Our study found that the Max VIS within 24 h after birth is an independent risk factor for poor early outcomes in EPIs (*P* < 0.05). Importantly, ROC curve analysis demonstrated that a Max VIS > 9.5 has good predictive performance for adverse outcomes, with an AUC of 0.680, sensitivity of 60.9%, and specificity of 74.9%. This suggests that dynamic monitoring of VIS changes can help identify high-risk infants early and adjust individualized hemodynamic support strategies, such as optimizing fluid management, necessary mechanical ventilation, and rational use of inotropic agents, to improve patient outcomes.

The MCA-RI is a vital indicator for evaluating cerebrovascular resistance and cerebral hemodynamics. Studies have shown that elevated anterior cerebral artery RI values correlate strongly with increased IVH risk (30). Potential mechanisms include immature cerebral autoregulation in EPIs, where high RI values indicate a state of high vascular resistance susceptible to blood pressure fluctuations, leading to cerebral hyperperfusion or ischemia-reperfusion injury. Reduced diastolic flow signifies unstable cerebral perfusion, increasing the risk of periventricular capillary rupture. Additionally, elevated RI values may often accompany systemic hypotension, heart failure, or infection, further aggravating brain injury (25, 31).

Our study found that elevated MCA-RI is a significant risk factor for adverse outcomes in EPIs (P < 0.05), likely due to excessive cerebral vasoconstriction and inadequate cerebral oxygenation leading to IVH. ROC curve analysis showed that an RI > 0.81 has predictive value for adverse outcomes, with an AUC of 0.693, sensitivity of 62.4%, and specificity of 72.7%. Thus, MCA-RI can serve as a key parameter for early identification of high-risk infants.

## 5 Conclusion

In summary, our study not only quantifies the challenges faced by EPIs at our center (high mortality and sIVH rates) but also identifies smaller gestational age, Max VIS, MCA-RI within 24 h after birth and vaginal delivery as significant risk factors for adverse early outcomes. Specifically, Max VIS > 9.5, MCA-RI > 0.81 within 24 h post-birth, vaginal delivery and smaller gestational age show good predictive performance for adverse outcomes. The combination of these four factors yields even higher predictive value, with an AUC of 0.833, sensitivity of 72.7%, and specificity of 81.4%. These findings provide objective, practical risk assessment tools for clinical practice. Therefore, careful consideration of delivery mode, close hemodynamic assessment with Max VIS calculation, and serial monitoring of MCA-RI within the first 24 h of life may be of great significance for the early identification of high-risk EPIs.

## Data Availability

The raw data supporting the conclusions of this article will be made available by the authors, without undue reservation.
